# Commentary: Predictions and the brain: how musical sounds become rewarding

**DOI:** 10.3389/fnhum.2017.00168

**Published:** 2017-04-05

**Authors:** Niels Chr. Hansen, Martin J. Dietz, Peter Vuust

**Affiliations:** ^1^Cognitive and Systematic Musicology Laboratory, School of Music, Ohio State UniversityColumbus, OH, USA; ^2^Center for Music in the Brain, Department of Clinical Medicine, Aarhus University and The Royal Academy of Music Aarhus/AalborgAarhus, Denmark; ^3^Center for Functionally Integrative Neuroscience, Department of Clinical Medicine, Aarhus UniversityAarhus, Denmark

**Keywords:** predictive coding, music, reward, pleasure, emotion, dopamine, neuroaesthetics

Converging research efforts have proposed that musical sounds become rewarding through predictive processes in the brain's pleasure networks, including dopamine release in the midbrain (Blood and Zatorre, 2001; Gebauer et al., 2012). In this commentary we address the subtle, yet important distinction between two types of “prediction error” that are sometimes conflated in the music neuroscience literature: (i) reward prediction error (RPE) pertaining to (psychological) expectations of how emotionally rewarding a piece of music will be and (ii) prediction error (PE) pertaining to neuronal computation of sensory input relating to the brain's predictions about music itself. Ultimately, “*What* is *the next chord?*” (PE) and “*How much will I* like *the next chord?*” (RPE) are distinct—potentially orthogonal—questions. While many sources of fundamental pleasure like food, sex, and drugs are readily quantifiable and show a largely monotonic relationship between stimulus amount and pleasure magnitude (until a given saturation point), sources of higher-order pleasures like music cannot be unambiguously quantified (Berridge and Kringelbach, 2008). More music does not in itself imply greater pleasure. Rather, the pleasure potential of music relies on the interplay of prior learning and dynamic changes in stimulus structure over time (Huron, 2006). We propose that predictive coding under the free-energy principle (Friston, 2009)—under which the brain continuously minimizes PE in the interaction with its environment—has the potential to bridge PE and RPE, thus elucidating domain-specific aspects of musical appreciation.

Salimpoor et al. (2015) take noteworthy first steps toward synthesizing the two research literatures on the neurobiology of reward (e.g., their references 2–9) and on musical expectations (e.g., their references 12–42). From a computational perspective, the former relies on reinforcement learning, which sets up computational principles for maximizing reward value, irrespective of music-structural specifics (Schultz, 2013). The latter deals with predictions concerning musical structure and has been modeled using statistical learning and predictive coding (Vuust et al., 2009; Hansen and Pearce, 2014; Vuust and Witek, 2014; Hansen et al., 2016). In predictive coding, PE is neither “positive” nor “negative” *per se*, but rather strong/weak on a single continuum (Friston and Stephan, 2007). Positive and negative RPE thus seems inconsistent with the mathematical formulation of predictive coding. Friston and colleagues propose that RPE represents mere surface manifestations of more fundamental computations in the brain (Friston et al., 2009). Specifically, rewarding actions are those that minimize the brain's free energy, thus building a stronger and more accurate model of the world. In other words, many types of reinforcement and procedural learning can be reinterpreted as predictive coding and may in fact render the very notion of value redundant (Friston, 2009).

A key claim of Salimpoor et al. (2015) is that “[w]hen listening to previously unheard music, similar-sounding auditory templates may be ‘activated’ to generate expectations of how the new sounds will unfold [i.e., PE]. If the new sounds were better than expected [i.e., RPE], positive PE would result.” Assessing whether music is “structurally-better-than-expected” requires a clear definition of “structurally good.” In music listening, however, expectations more likely pertain to the structure of music (PE) than to its reward value (RPE) (Huron, 2006; Miranda and Ullman, 2007; Hansen and Pearce, 2014; Vuust and Witek, 2014; Hansen et al., 2016). Accordingly, Salimpoor et al.'s notion of valenced PE with respect to structural continuation is problematic because it conflates expectations about experienced pleasure and perceived sounds. Yet, the authors resort to this in their account of the inverted U-shaped relationship between exposure and musical appreciation, claiming that time between hearings increases the leeway for positive PE (Salimpoor et al., 2015, Box 2). In Figure 1 we provide an alternative explanation, without reference to RPE, emphasizing instead how the certainty of the brain's predictions influences the salience of the ensuing PE (Ross and Hansen, 2016) which may in turn affect the level of experienced pleasure. This is thought to be mediated by the sensitivity or gain of neuronal populations in inferior frontal gyrus to feedforward connections from superior temporal gyrus that mediate PE (Dietz et al., 2014).

**Figure 1:**
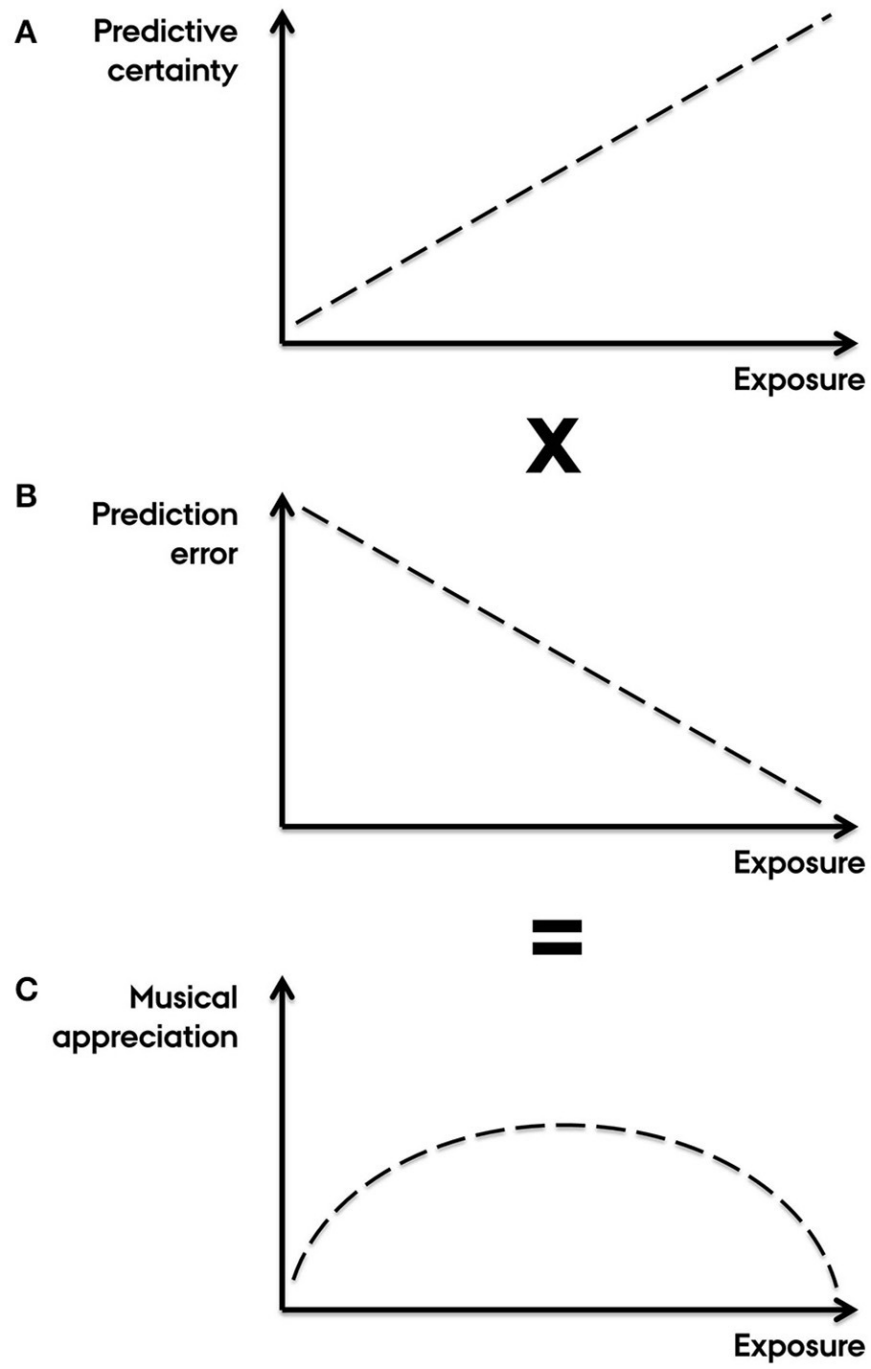
**Predictive coding of musical appreciation with increasing exposure**. Understanding the inverted U-curved relationship between exposure and appreciation is a key question in the cognitive neuroscience of music (Salimpoor et al., 2015). Here, it is unadvisable to conflate predictions about musical structure (PE) and its reward value (RPE) (cf. Salimpoor et al., 2015, Box 2). The alternative account presented here is based purely on PE, with no reference to RPE or conflation of the two. Instead, we emphasize contrastive interactions between *schematic expectations*, pertaining to generalized knowledge about a musical style, and *veridical expectations*, pertaining to specific knowledge about particular pieces (Huron, 2006). These two types of expectations have distinct neural representations (Miranda and Ullman, 2007). **(A)** Initially, because unfamiliar music affords a weak predictive model, the gain on error units is set such that prediction error is low in salience (Friston, 2009). With increasing exposure, music is contextualized (e.g., determining key, tempo, meter, instrumentation, duration, form, genre) leading to schematic expectations with higher certainty (Hansen and Pearce, 2014; Hansen et al., 2016). Simultaneously, veridical expectations arise causing potential conflicts with schematic expectations (Huron, 2006). This sharpens the listener's predictive model generating stronger expectations with higher error-unit gain, leading to gradually more salient prediction error (Hansen and Pearce, 2014; Hansen et al., 2016). **(B)**. With increasing levels of exposure (and ultimately over-exposure), the amount of prediction error gradually declines as veridical expectations become increasingly aligned with sensory input and thus are assigned greater relative importance compared to schematic expectations. **(C)** The combination of increasing certainty-weighting of prediction error (due to gradually higher predictive certainty) and fewer instances of prediction error (due to minimization of free energy) results in an inverted U-shaped trajectory of musical appreciation with increasing levels of exposure. This is consistent with dopamine coding for the precision of prediction error.

Previous studies have found a relationship between reward value and activity in brain structures implicated in positive RPE (Salimpoor et al., 2013). This does, however, not provide causal evidence that musical appreciation is mediated by positive RPE, rather than PE. Moreover, a general focus on RPE circumvents the question of how music evokes pleasure and is assigned reward value in the first place.

So how does dopamine fit into this? Single-cell studies have shown bidirectional coding where changes in dopaminergic activity reflect positive RPE when a reward is greater than expected and negative RPE when it is smaller than expected (Schultz, 2010, 2013). However, empirical evidence indicates that dopamine neurons not only code for expected reward value, but also for the magnitude, timing, probability, and uncertainty of rewards, as well as perceptual salience (Schultz, 2010; Vuust and Kringelbach, 2010). The last possibility, in particular, may relate to PE rather than RPE. Rather than encoding the “prediction error on value,” predictive coding posits that dopamine may encode the “value of prediction error” where value corresponds to precision or incentive salience (Friston and Stephan, 2007; Friston, 2009). For example, one may hypothesize that participants are willing to pay more money for music that they have a strong predictive model for (cf. Salimpoor et al., 2013).

In conclusion, we propose that predictive coding offers a useful framework for understanding the mechanisms that determine when and why music is rewarding. However, it is crucial not to conflate RPE and PE. We regard this as an important distinction that still remains to be adequately studied. To this end, although not the only relevant theory, predictive coding could provide an alternative account of musical reward encapsulated in a general theory of brain function where PE and RPE are treated in a unified manner. In other words, predictive coding of musical structure and its rewarding qualities may be different manifestations of the same underlying computational principles.

## Author contributions

NH and MD: conceived of the initial idea for this commentary. NH: wrote the first draft. NH, MD, and PV: all made substantial contributions to the content, critical revision, and final approval of this work and agree to be held accountable for this.

## Funding

Center for Music in the Brain is funded by the Danish National Research Foundation (DNRF117). During part of this work, NH was supported by The Ministry of Culture Denmark and an EliteForsk Travel Grant from The Ministry of Higher Education and Science Denmark. MD is supported by the VELUX Foundation.

### Conflict of interest statement

The authors declare that the research was conducted in the absence of any commercial or financial relationships that could be construed as a potential conflict of interest.
